# The Scope of Pharmacological and Clinical Effects of Modern Antihistamines, With a Special Focus on Rupatadine: *Proceedings from a Satellite Symposium held at the 21st World Allergy Congress, Buenos Aires, December 8, 2009*

**DOI:** 10.1186/1939-4551-3-S1-S1

**Published:** 2010-04-15

**Authors:** Martin K Church, Jorge F Máspero, Marcus Maurer, Dermot Ryan, G Walter Canonica, Carlos E Baena-Cagnani

**Affiliations:** 1 Inmunopharmacology School of Medicine of the University Southamptom, UK; 2 Head Pediatric Allergy, Deutsche Hospital, Buenos Aires, Argentina; 3 Dermatology and Allergy, Hospital Charite, Berlin, Germany; 4 Clinical Research Fellow, University of Aberdeen, United Kingdon; 5 Allergy & Respiratory Diseases Clinic, Genoa University, Italy; 6 Faculty Medicine Catholic University Cordoba, Argentina

## Note

Consult PDF for full article

